# Human capital space: a spatial perspective of the dynamics of people and economic relationships

**DOI:** 10.1057/s41599-023-01639-5

**Published:** 2023-04-06

**Authors:** Zhenshan Yang

**Affiliations:** 1 Institute of Geographical Sciences and Natural Resources Research, Chinese Academy of Sciences, 100101 Beijing, China; 2 University of Chinese Academy of Sciences, 100039 Beijing, China

**Keywords:** Geography, Development studies, Education, Economics

## Abstract

While researchers increasingly recognise drastic changes in populations and repeatedly emphasise their implications for development, far less attention is devoted to thinking of and making spaces available for people. This article proposes the concept of human capital space (HCS) and elaborates on its typology, spatial externalities, selection-sorting-matching mechanism, and crucial role in building dynamic capabilities in cities and regions. Theoretical discourses and constructs furnish reasons to believe that HCS is a useful instrument to examine the complex people–space relationship and to encourage conversations about the interactions among population, labour, economic geographies, and related disciplines. HCS provides a terrain for scientists to actively engage in human-centred spatial development, inform policies in a timely manner, and argue for effective investment in space to bolster the endogenous power of spatial development.

## Introduction

History is replete with processes of capitalisation of people and spaces. However, over the past 50 years, spatial emphasis has been heavily skewed towards economic issues, with substantial land developed for economic ends, and significant research has focused on the distribution and organisation of economic activities. Although society and space are mutually constituted (Lefebvre, 1991), and we have dramatically improved our knowledge on how space is developed to facilitate economic growth (Thrift and French, 2002; Werner, 2021), we know little about how people grow by using space.

Besides wealth creation, people also need healthcare, recreation, social networking, education, skill training, and other supportive activities. The availability of these services is dependent on investment in space. Some of these activities are the main components of household economies and the modern economy (Tickell, 1999; Tickell, 2002; Smith and Stenning, 2006; Hall and Page, 2009; He et al., 2020) while others are critical public concerns (Kearns and Moon, 2002; Winter, 2012; Rosenberg, 2016) such as healthcare. In the context of COVID-19, safe, convenient, and well-equipped healthcare facilities are more favourable for people’s lives (Finn and Kobayashi, 2020) and for countries and regions to maintain their growth. However, there is a deficit in investment in schools and healthcare facilities, which directly serve human development. The investment type is to a large extent a ‘patient capital’, and governments are caught between concerns for public welfare and economic returns. As Marshall (1890: p. 564) claims, ‘the most valuable of all capital is that invested in human beings’. There is a dire need to consider investment for human development and new ideas to understand the spatial implications of people and economic relationships (PERs).

There is a continuous call for attention to the role of the population in an economy, with talent (Florida, 2002; Faulconbridge et al., 2009; Nifo and Vecchione, 2014; Geddie, 2015; Yang and Pan, 2020b; Adler and Florida, 2021; Gu et al., 2021; Raghuram, 2021; Cui et al., 2022) and creative classes (Florida, 2005; Asheim and Hansen, 2009; Lorenzen and Andersen, 2009; Alfken et al., 2015; Audretsch and Belitski, 2021; Bergan et al., 2021) in knowledge economies (Asheim et al., 2007; Mudambi, 2008; Rantisi and Leslie, 2010; Cicerone et al., 2021; De Propris and Bailey, 2021). While globalisation has dramatically expanded the space for production (Coe, 2011; Werner, 2019), the returns on the factors of production decrease with a product surplus or competition among homogeneous products (Adhikari and Paul, 2018). It is increasingly difficult to maintain economic growth by relying solely on production space or land expansion without continuous inputs of knowledge and technology to invent and produce new and high-quality goods and services (Cooke, 2005; Rausch and Negrey, 2006). An accumulation of talent becomes critical in breaking the bottleneck and forging new paths for the spatial economy (Qian, 2017; Yang and Pan, 2020a). That is, the quality of people is (will be) vital for current (future) urban and regional growth.

In addition, the number of people matters in spatial development. Spatial development can be understood as space transformation in relation to the protection, enhancement, use, management (Ministry of Sustainable Development and Tourism, 2013), and socioeconomic growth of a defined area. Many cities and regions face uneven population flows and contractions (Oswalt and Rieniets, 2006). In 2019, for the first time, people older than 65 years exceeded children younger than 5 years in number worldwide (United Nations, 2019). In the United States, Europe, Japan, and China, fertility declines with economic development (Jarzebski et al., 2021). Population decline may profoundly affect local land use, social welfare, and the fiscal system through the complex interactions among production, social welfare distribution, and the use of those fiscal systems (Yang and Dunford, 2018), along with vacant properties and land, labour force shortages, and the interplay of economic decline and fiscal austerity (Großmann et al., 2013). Therefore, population decline may make spatial development in certain areas risky.

Accordingly, this study explores the sophisticated spatial dynamics of PERs. It adopts the concept of human capital (HC) and extends it to spatial discourse to capture the importance of humans in spatial development. HC refers to people’s knowledge, technology, experiences, health status, and mobility. In addition to quantitative characteristics, such as population decline, population quality influences spatial development, particularly due to the increasing importance of knowledge and technology in economies. Furthermore, people with higher HC tend to be well paid, driving the consumer economy (Florida et al., 2008). As different people work and live in different places, HC is spontaneously distributed at an uneven pace. Places with high HC can attract more talent, who expect to be able to share ideas and knowledge and enjoy their working habits and lifestyles (Ewers, 2007; Su et al., 2020). Moreover, this provides employment opportunities for other people due to linkages among firms and economic activities (Díaz, 2013).

Broadly, there are several major material and immaterial powers of wealth production: natural capital or wealth, including land, minerals, climate, plants, and animals (Shao and Yang, 2014); physical capital, including tools, machinery, and infrastructure (Lopez-Bazo and Moreno, 2008); HC, which recognises the economic value of a worker’s education, skills, dexterity, intelligence, health, and leadership and personality attributes as capital input into economic processes (Schultz, 1961; Romer, 1990); and social capital, which regards the ability to build social networks or relationships in society (Currid-Halkett and Ravid, 2012). In a given area, these types of capital are strongly correlated. For instance, HC accumulation stems from using natural and physical capital and can be reinforced by social capital (Currid-Halkett and Ravid, 2012). Together, they serve as powers to promote spatial development. The continuous use of natural capital and the advancement of physical capital largely hinge on the HC level. However, HC cannot play this role without other types of capital such as social capital (Tsuda, 2011; Currid-Halkett and Ravid, 2012).

HC integrates the outcomes and drivers of human development through investment in education, skills, and healthcare. It promotes knowledge about the value and benefit of places of residence and work. As Bourdieu (1986: p. 242) said, ‘It is in fact impossible to account for the structure and functioning of the social world unless one reintroduces capital in all its forms’. HC clarifies the spatial development structure and function of the social world. Meanwhile, it enhances the well-being of people and their generations through feedback loops of economic progress and improvements in education and healthcare (Eker and Ilmola-Sheppard, 2020). As HC is regarded as an endogenous power of economic growth, it can also act as an endogenous driver of spatial development. Exploring this topic can furnish insights into existing and previous spatial differences and dynamics.

Therefore, HC is a factor, driver, and outcome of spatial development. Accordingly, it is reasonable for geographical studies to investigate PERs, as people grow and the economy develops in space, which are linked by the HC concept. Notably, the traditional understanding of HC stems mainly from the economic and demographic fields and primarily is concerned with certain qualitative attributes of people such as education and skills. Nonetheless, this study provides a spatial perspective to expand on the concept of HC. To this end, it investigates the spatial differences of HC and the associated geo-settings; it thus facilitates a spatial understanding of PERs, which also helps establish a link among the prevailing concepts of population, labour, and talent in geographical investigations.

The novelty of this study is demonstrated in its proposal of the concept of human capital space (HCS) for an in-depth consideration of spatial representations and the implications of PERs. Spaces such as schools, hospitals, and amenity facilities are important for human development; however, in many cities and regions, there is a lack of investment into these factors. We argue that human and spatial development are reciprocal. In addition, during a ‘place-war’ for talent, many regions focus on economic value, ignoring people’s social needs and demands for education, training, and skill improvement. Therefore, current spatial development reaps the harvest of human development rather than being formed based on human development. As people move between spaces, this makes long-term spatial development risky, especially during population shrinkage. Existing studies include approaches from fields such as economic geography, population geography, economics, and management and have contributed a significant amount of evidence, ideas, and thoughts. However, previous studies are limited to specific portions or episodes of the relationship between people and the economy in a given space, such as economic geography focusing on amenity and talent or demography and economy focusing on investment and economic rewards. Therefore, our study aims to consider mutual effects between humans and spaces through investment and rewards to encourage cross-disciplinary research. By focusing on space, we learn numerous ideas from economic geographers such as Florida (2005; 2015), and Storper and Scott (Storper and Scott, 2009). However, geographers mainly focus on drivers of the spatial movement of talent (amenities) and their spatial impacts with little attention given to the definition, types, levels, and quality of talent or how to cultivate that talent. Therefore, we argue that some questions remain unanswered. Moreover, through cross-disciplinary learning, we hope to expand the view of economic geography to further examine the knowledge, people, and economy in space and make it more applicable to real-world issues.

This study argues that HC is a useful concept when exploring PERs in space and that space is an agent that should be incorporated into the HC concept to explore the interaction between people and space and the reciprocity of investment and rewards for people and space (section ‘Capturing the relationship between people and the economy based on human capital in space’). HCS is depicted by a typology of spaces for HC accumulation, substantiating the linkage between human and spatial development (section ‘Spaces of human capital accumulation’). Section ‘Human capital: spatial externality and endogenous drivers of spatial development’ discusses spatial externalities to explain how HC can endogenously drive spatial development. The heterogeneity of HCS can be explored by a selection-sorting-matching mechanism, enabling analysts and practitioners to probe the reasons for the interplay between people and space (section ‘Human capital in space: selection, sorting, and matching’). In addition to the rewards for people and space, the study argues that HC attracts and leverages external resources, which offers a region dynamic and lasting power for development (section ‘Dynamic capabilities and investment’). Finally, section ‘Conclusions’ recaps the main findings and concludes that spatial investment in humans should be given more attention.

## Capturing the relationship between people and the economy based on human capital in space

HC is intangible but embodied by people in a given space. It includes people’s income, education, skills, intelligence, and health status. The concept was co-developed by several schools; this warrants pondering over its value in promoting development and its spatial relevance, which requires further exploration. Economic approaches regard HC as a form of capital in economic development, and accordingly, emphasise the significance of investment in labour, with a primary focus on education and health (Schultz, 1961; Romer, 1990). Business management frequently uses the concept of HC in firms’ search for profit by encouraging learning, innovation, and organisational optimisation (Nyberg and Wright, 2015; Gerhart and Feng, 2021). Both approaches focus on the HC of workers and neither examines how HC differs spatially. They largely fail to incorporate HC when explaining the location and distribution of economic activities. As an asset of space, HC relates not only to labour but also to people. People with high educational attainment may leave their hometown if they cannot find employment; thus, their hometown loses HC.

HC connects and complements population, labour, and economic geography research and is more actively present in spatial economic development. Population geography largely focuses on the distribution and movement of people and regulates them as dependent variables of spatial dynamics by emphasising the quantity aspect of population, which is insufficient to describe the demographic attributes of a certain place (Rogers, 2008; Bailey, 2010). In contrast, qualitative attributes, such as income, education, and health, are more frequently associated with economic levels of development and therefore link humans and economic development dynamics. Labour geography claims that workers are a distinct, autonomous force in particular temporal and spatial circumstances (Siemiatycki, 2012). Recent studies of labour geography have devoted much effort to examining workers’ actions, including migration and labour agency, in certain political systems in response to societal or capitalist changes (Castree, 2007; Coe, 2013; Dutta, 2016). However, they fail to treat humans explicitly as a form of capital or explore the formation of labour quality. Mitchell (2005: p. 59) points out that labour geography typically focuses on employment. There is also some communication between population and labour geographies, and population movement can be regarded as a redistribution of labour in space (Fan, 2005) and labour’s spatial ‘fix’ (Herod, 1997). Economic geography examines economic activities across space and explains the spatial economic dynamics of cities and regions (Baldwin and Okubo, 2006; Storper and Scott, 2009). A growing body of related studies argues that knowledge economies are shaping and driving economic landscape transformations (Tether et al., 2012; Jacobs et al., 2014; Marchand et al., 2020). Moreover, economic geographers are interested in studying the impacts of talent, the creative class, and amenities on cities and regions (Florida, 2002; Florida, 2005; Lorenzen and Andersen, 2009; Storper and Scott, 2009). Such studies provide a means to examine the flow of HC in the context of the movement of people and labour.

**Fig. 1:**
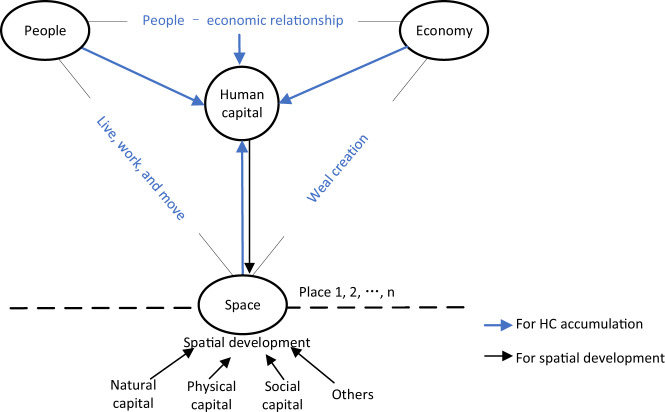
Human capital and space in societal development. People, the economy, and space are the three main dimensions in societal development. The figure depicts the interactions and relations among them. Human capital (HC) captures people’s and economic relationships, whose accumulation depends on favourable relations and spatial settings such as natural resources, infrastructure, and facilities. Space is developed by human, natural, physical, and social capital and others. However, places are intertwined and compete with each other. Therefore, HC flows in space with people’s movements.

The abovementioned studies lay a strong foundation for understanding the value of humans to the economy and how and why people or workers float in space (Fig. 1), reflecting the complex PERs in different places. However, many questions remain unanswered: Why do some areas have higher HC than others? How can areas be made more attractive to people to achieve higher HC? What type of HC do areas aim to increase? Can cities and regions cultivate HC by themselves beyond attracting talent, and how can they retain it? These questions have much theoretical and practical value in the current knowledge-oriented economy and place-competition era. For instance, the Rust Belt to Sun Belt migration continues in the US (Ceh and Gatrell, 2006; Glaeser and Tobio, 2007). Likewise, in China, a troop of college graduates flow into coastal areas; even the proportion of graduates from Peking and Tsinghua Universities staying in Beijing decreased dramatically from 72 and 31% in 2013 to 16 and 18% in 2019, respectively (Sina Finance, 2021). The underlying reasons are complicated and extend beyond the economic issues of jobs and wages affecting living standards, costs, and natural amenities. Apparently, these factors, even the economic ones, have spatial attributes.

People, the economy, and space comprise the three main dimensions in societal development (Fig. 1). Relative to the other approaches noted, it is important to investigate the relations and interactions between HC and space for two reasons. First, HC captures PERs because it offers an endogenous power of economic growth and enhances the well-being of people. Second, spaces are agents of change (Oblinger, 2006), and changing spaces will change PERs. This model has important implications (Fig. 1). Theoretically, it integrates people and the economy, with an outlet to other dimensions, such as physical and natural dimensions in societal development, which is much closer to reality. Practically, it enables practitioners to ponder spatial engagement, including investment in prioritising human development rather than the economy alone.

This study adopts this model to incorporate space into the HC concept. Traditionally, HC is regarded as a co-result of demographic and economic changes, largely overlooking the role of space. People and space interact through the reciprocity of investment and reward. This reciprocity exists in various human activities such as education, production, consumption, and recreation and is upgraded and augmented, increasing in sophistication as people’s abilities enhance. Reciprocity in HC investment and rewards is well analysed in the economics literature, primarily through differentials between education and income (Mincer, 1958; Schultz, 1961; Romer and Barro, 1990). However, space profoundly induces people to enhance their HC via facilities such as schools, training centres, and healthcare facilities (the next section provides more details).

Thus, HC constitutes the continual momentum of spatial development. Its changes in a place imply PER changes, largely reflecting and affecting its social and economic development. A place is usually prosperous when people and the economy can positively influence one another as in metropolitan areas (i.e., industries and people can attract one another). In contrast, a place most likely encounters socioeconomic problems given tense conditions such as high unemployment and unattractiveness from the perspective of firms because of the lack of suitable labour. As HC furnishes insights into PERs, rather than merely people or the economy, it is meaningful to consider spatial development. Thus, analysts and practitioners can consider examining PERs and expending more effort on enhancing facilities for human development. With the augmentation of and investment in individuals’ HC, associated places gain more power and choices to generate higher social and economic rewards.

Therefore, a spatial approach to HC is required that focuses on the geographical differences in and structure of HC, the role of space influencing peoples’ HC, and the leveraging of HC in promoting spatial development. Unlike economics and business management approaches that primarily focus on the economic profits of HC, the spatial approach emphasises the reciprocity and interaction between people and spaces. It provides opportunities to incorporate a more active sense of humans as geographical agents into the dynamics of space, assisting to ‘theorise how workers (people) attempt to make space as an integral part of their social existence (in labour geography)’ and filling in the gap of ‘writ(ing) less capital-oriented (human and) economic geographies’ (Herod, 1997).

HC in geographical explorations juxtaposes the quantitative and qualitative features of and changes in people in a spatial context, which evolves, especially qualitatively, with socioeconomic progress in society. Measuring HC in space is challenging. Empirically, educational attainment is often used as a proxy, as it is highly correlated with technological innovation, economic outputs, and personal income (Mincer, 1958; Mincer, 1989). This measurement has been refined by assuming a linear relationship between years of schooling and the level of HC (Barro and Lee, 1993). However, it still only measures the role of education in forming HC without considering other factors (Roca and Puga, 2016). A lifetime income approach has been proposed to calculate HC as the present value of expected future lifetime earnings in five stages: work only, work–school, school only, pre-school, and retirement (Jorgenson and Fraumeni, 1992; Li et al., 2014). This measurement’s value lies in including all aspects of HC measured by market value; further, as the calculation process is more analogous to physical capital, it allows for a better comparison. However, because of the ease of data collection and availability, the educational method dominates the current literature, as the lifetime income approach requires both macro- and micro-data, which are difficult to acquire. Overall, there is a dire need for innovative and practical approaches to examine the connotations of HC, particularly in geographical studies.

## Spaces of human capital accumulation

Spatial and human development are reciprocal and intertwined but not always simultaneously or synchronously because of human–spatial movement. This process gives rise to spatial heterogeneity in HCS, where some areas accumulate a higher level of HC than others on a given spatial scale. Bourdieu (1986) argues that the distribution structure of different types of capital at a given moment in time represents the immanent structure of the social world, which governs its functioning and determines the chances of success for practices in the real-world. Similarly, HCS provides a lens for understanding the pattern, process, and mechanism behind the spatial representations and dynamics of PERs; substantiates the means to invest; and promotes spaces to foster human development and spatial growth.

There are three types of HCSs, informed by lines of thought on the movement of people, talent and labour, and economic and social development: HC employment space (HCES), HC cultivation space (HCCS), and HC refreshing space (HCRS).

### Type 1: HCES

HCES is a place where people acquire skills and use their HC to create value for society. The idea is closely associated with the evolution of industrial space and the clustering of talent, embodying skills, professional experiences, and innovative intelligence. In employment space, HC is similar to the concept of intellectual capital ‘packaged as useful knowledge’ (Sveiby, 1997) in the value-creation processes of enterprises (Grasenick and Low, 2004; Tandon et al., 2016) and talent-related studies in the geographical literature (Florida et al., 2012).

HCES coincides with places with locational attractions for firms and people and is unevenly distributed in space. By examining computer services in southeast England, Coe and Townsend (1998) investigated the ‘myth of localised agglomeration’, showing that, despite inter-firm linkages across relatively large geographical distances, localised concentrations of firms still exist. This indicates that HCES may be concentrated in some areas but has citywide or regional effects that attract related firms and services. HCES is critical to maintaining the vitality and competitiveness of cities and regions in the globalisation era.

Further, HCES provides space for on-the-job skill accumulation (Hansen and Imrohoroglu, 2009), where employees can acquire skills through learning by doing. It is a critical step for people to continue to be trained to master the knowledge and skills required for the job. In modern economies, pre-job or on-the-job training is prevalent. For some industries (e.g., banking), training is frequent. Vocational training is organised by firms, associations of firms, and local governments to disseminate and upgrade knowledge regarding HC required for local industrial upgrading or restructuring and create lifelong learning strategies for people to accumulate HC (Tsang, 1997; Cort, 2009).

Many contextualised reasons explain the formation of and changes in HCES. In China, the apprentice-based learning system was established when industrialisation was in its infancy (Zhu et al., 2016). Although the industrial structure is often conceived as the main factor affecting the labour training system, the apprenticeship system in the British construction industry has declined, whereas it has survived in Australia. The contrasting results stem from the differences in institutions, organisation of employers and labour, and training systems (Toner, 2008). Globalisation speeds up the development of HCES, which is a synthesised space of a firm’s internal labour-management relations and inter-organisational relations (Zhu et al., 2017), to foster the exchange of local and external knowledge.

### Type 2: HCCS

HCCS is the place where people receive an education, mainly primary and high schools, universities, and research institutes. The first is a sub-type of education for children and is usually a choice made by families (Kromydas, 2017) that has dual implications. First, cultivating HC affects present and future levels of knowledge and technology. Second, the location of primary and high schools, especially good schools, affects the residential choices of families and results in so-called *jiaoyufication*, which becomes a force of gentrification and middle-class makeover in cities (Wu et al., 2016; Yang et al., 2018). Within spatially limited school catchment areas, *jiaoyufication* narrows opportunities for intergenerational social mobility and exacerbates social polarisation (Wu et al., 2018; Hu et al., 2019), which makes this type of HCCS a social space with the power to gradually replace traditional social hierarchies and perhaps establish neoliberal stratification.

The second sub-type focuses on higher and professional education that prepares students more directly with the necessary knowledge, skills, and technology for the job market. Its role was first addressed to promote economic growth and the call for a synergy between universities and industries to enhance industrial innovation; this sub-type appeared later in the studentification literature (He, 2015; Nakazawa, 2020), which was interested in urban socio-spatial transformation triggered by an increase in and concentration of student populations. While previous research endorsed university–industry collaboration for promoting knowledge spillovers from academic research to regional innovation (Miyata and Shavinina, 2003; Ponds et al., 2010; Eerola et al., 2015), recent studies in the United States show that universities appear to primarily create HC rather than knowledge spillovers for nearby firms (Fallah et al., 2014). The reasons for this may include low technical cooperation between universities and firms, university training not being updated in line with the requirements of firms, and graduates being employed nearby but not engaged in the sector they trained for. HC and knowledge spillovers contribute differently to firms because the former is a type of capital and the latter is kind of knowledge or skill for innovation. Given the insufficiency of academic education on vocational skills training, firms may actively participate in college curriculum design; accordingly, public and private vocational colleges have been established in industrial design firms in Beijing (Zhu and Li, 2019).

It is worth examining the role of universities through further empirical investigation rather than simply accepting the phenomenon that universities and innovative firms are proximal or simply calling this ‘university-linked knowledge spillovers’ (Feser, 2002). Nevertheless, with geographically bounded knowledge spillovers (Ponds et al., 2010) or distance decay (Feser, 2002; Rammer et al., 2020), this type of HCCS compels the spatial heterogeneity of learning and technological divergence (Menzel and Fornahl, 2010) with a city or region but builds up icons for the city as higher education centres and college towns (Ehlenz and Mawhorter, 2021). Studentification literature rightly covers a wider spatial influence of this type of HCCS. However, further efforts are required to explore the diversity and mobility of studentification, as college students have a strong inclination towards movement. Studentification has far-reaching effects on HC level and spatial development, as new workers drive the population re-production of a place.

### Type 3: HCRS

HCRS primarily aims to meet people’s social needs and covers a range of amenities and services, including climate amenities, friendly neighbourhoods, consumer and recreational spaces, and healthcare services. The first sub-type refers to climate amenities—examples include the attraction and growth of Sun Belt cities in the United States (Glaeser and Tobio, 2007) and the continual loss of population in the colder north-eastern regions of China (Yang, 2019). Air pollution also adversely affects older adults and less-educated migrants, who account for a large proportion of the urban labour force in China (Liu and Yu, 2020).

The second sub-type refers to studies of neighbourhoods. Community studies are increasingly interested in testing the effect of the built environment on life satisfaction and well-being and include various neighbourhood characteristics, such as walkability, transit, parks (Pfeiffer et al., 2020), and educational amenities (Patacchini and Zenou, 2011; Midouhas et al., 2014). The third sub-type refers to consumer and recreational spaces. Studies show that leisure amenities attract people, especially highly skilled workers, by providing diversified entertainment opportunities, cultural and sports facilities, and high-quality restaurants (Saiz et al., 2001; Glaeser and Gottlieb, 2006; Carlino and Saiz, 2019). This type of urban growth based on people’s demands impacts contemporary cities and the regional spatial structure (Burger et al., 2014; Lanzara and Minerva, 2019). The fourth sub-type refers to space for healthcare. Although there is limited literature on human and economic geography dedicated to the relationship between healthcare and HC as well as between healthcare and the quality of a place, it has received more attention since the COVID-19 pandemic. Darlington-Pollock and Peters (2021) studied ‘health-selective migration’ to enhance our understanding of the new mobilities paradigm but failed to provide insights into how it affects human and spatial development. A contrast exists between the demands on public health services and the insufficient supply of healthcare services. Socioeconomic infringements during the pandemic may have significantly undermined HC accumulation in both quantitative and qualitative terms.

**Fig. 2:**
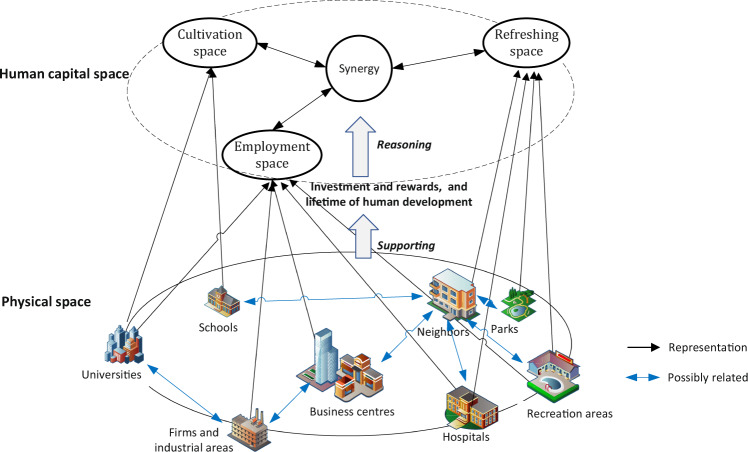
Human capital space: Linking human and spatial development. The three types of human capital spaces (HCSs) are illustrated by physical entities. Their synergies can be explained by the reciprocity of investment and rewards during the lifetime of human development. Through the projection of HCS in physical spaces and complex relations among physical entities, HCS explains human-centred spatial development.

These three primary types of HCSs are organically linked as HC augmentations and rewards that span a lifetime (Fig. 2). For people, HCCS helps cultivate HC, HCES helps acquire further skills and reflect the value of HC, and HCRS provides occasions for people to enjoy the benefits of their HC enhancement. Regarding space, such as cities, HCCS helps educate people in schools and universities, HCES helps utilise HC to harvest the value of internal and external HC, and HCRS rewards HC utilisation and facilitates sustainable use of HC. The multiple meanings of HCS connect human and spatial development, putting people at the heart of the concept as society progresses towards human development, with space restructured accordingly.

HCSs are organically connected to reflect the interaction and interplay of people and the economy. For instance, the linkage between universities and industry spin-offs fosters knowledge incubation and knowledge economies (Fallah et al., 2014); mutual promotion exists for the creative class and the ‘quality of place’ (Trip, 2007) or ‘power of place’ (Florida, 2002). The creative class describes people employed in occupations such as sciences, engineering, education, culture, arts, and entertainment; such people are expected to live in comfortable environments and socially favourable places (Florida, 2005). In US metropolises, there are positive associations between cognitive, technical, problem solving, social, and managerial skills and high-technology start-up activity, and these skills play moderating roles in turning university research into entrepreneurial activity to consolidate knowledge-based regional economies (Qian, 2017). Currid-Halkett and Ravid (2012) affirm HC mobility in the cultural industry and its impact on places, showing the connectivity between these places. These studies reveal the amalgamation of employment, cultivation, and refreshing spaces of HC, which is attributed to people’s innate needs.

Appreciating the connection and interaction of HCSs may offer new insights into people and spatial dynamics. For instance, Glaeser (2011) holds that amenities induce the flows of people and stimulate urban growth. Storper and Scott (2009) argue that the amenity-driven approach is ill-advised and that there are complex recursive interactions between firms’ locations and labour movements. However, HCS encourages the examination of spatial development oriented with human development, juxtaposing amenities (HCRS), jobs (HCES), and schools and universities (HCCS). Accordingly, beyond discussing the enigmas of ‘do jobs follow people or do people follow jobs’ (Storper and Scott, 2009), this study explores how to create spaces for people. Thus, amenities are part of the drivers of spatial development but are better connected with other HCSs. The amenity-driven approach makes sense because it reveals that lifestyle influences people’s migration and movement, as per Storper and Scott (2009), who posit that jobs and income are basic needs of people. However, education has become an additional influential factor, as the *jiaoyufication* approach indicates. Reportedly, citizens in Beijing choose their residences by striking a balance between the proximity to jobs and their children’s schools rather than solely considering the former (Yang et al., 2018).

Spatially, the formation and impacts of HCS must be examined to understand a region, city, or neighbourhood, as people’s activities, facilities, and services have a certain geographical reach. For instance, in Berlin, a microgeographical scope of about 50–250 m exists for knowledge sources and industrial innovation in urban environments (Rammer et al., 2020). The epicentre is manifested in several areas because of the uneven spatial process of HC flows and, more importantly, the space as a supportive player, but has great spillover effects on industrial activities for employment and overall city branding for education and amenities. Consequently, the development of these spaces may impact certain city characteristics. For instance, education cities (Kleibert et al., 2021) and college towns (Ehlenz and Mawhorter, 2021) attract talent who choose their residence and other activity spaces in the city.

## Human capital: spatial externality and endogenous drivers of spatial development

HC offers an endogenous power for development, and so does space. Arguably, most geographical models tend to rely on exogenous factors to stimulate spatial development, such as regional multipliers and trades (Crevoisier and Rime, 2021). In the globalisation era that started in the 1980s, the theories of global production networks (Yeung and Coe, 2015) and industrial clusters (Porter, 1998; Fujita et al., 2001) predominated the organisation of local spatial models by noting competitiveness, embeddedness, networking, division of labour, and production. There is a large gap in the balance and synergy of endogenous and exogenous factors in spatial development. HC sustains the momentum of economic growth through knowledge and technological innovation. Schultz (1961) posits that HC is the most distinctive feature of the economic system by observing that skills and knowledge investments constitute a form of capital that grows faster than non-HC when economies are in the development phase. HC is an engine of productivity and growth through innovation and the adoption of technology (Romer, 1990; Aghion and Howitt, 1992; Danquah and Amankwah-Amoah, 2017). Its level greatly affects the ability of countries and regions to develop technological innovations and disseminate knowledge (The World Bank, 1998; Florida et al., 2012; Marrocu et al., 2013). In the globalisation era, HC is a crucial factor to facilitate technological progress in developing countries (Li et al., 2014). HC helps China absorb foreign investment and invest abroad (Yang et al., 2021). The successive exploration of the endogenous growth theory stimulates a shift in attention from physical capital only to incorporating HC, thus developing an in-depth understanding of wealth creation and distribution and intergenerational changes in society.

In addition to impacts that improve productivity in production and enhance income for individuals, geographical and economic studies have identified spatial externalities as having incidental impacts on HC accumulation. These processes happen through the reciprocation and feedback among HC accumulation, economic growth, movement of people, spatial spillover, and the improvement of public services.

First, HC becomes a spatial identity of place, such as the ‘quality of place’ in Amsterdam and Rotterdam (Trip, 2007). Consistently, scholars suggest that the policymaking process should be carefully designed to attract and retain talented and highly educated workers (Lepawsky et al., 2010) by combining both contextual and spatial elements in the liberal market economy around mobility, adjustment, and quality of place (Clifton et al., 2013).

Second, HCS increases the value of an area. In the housing market, HCS is often seen as a housing premium because highly paid workers are willing to and can spend more for a shorter commuting distance. Therefore, the housing market is more directly related to the spatial externality of HC rather than proximity to industrial areas. Land in the HCCS also has a premium based on its proximity to good schools within a short commuting distance (Cannon et al., 2015). Housing prices are commonly significantly higher in large cities such as Beijing, London, and Vancouver. This dramatically increases costs, but by no means contributes to HC acquirement and accumulation, although value is added to adjacent areas.

Third, HCS benefits people and labour in adjacent areas. Some people with no direct links to HC in the area and surrounding areas could benefit from the promotion of HC through more job opportunities, better living environs, higher social status, and rising real estate prices. Ehrl and Monasterio (2021) show that the spatial concentration of analytical skills generates positive wage externalities for all workers in the local labour market, and this externality is independent of the classical market size economies.

Fourth, HCS attracts population flows to an entire city or region. This trend is more salient in the location–globalisation interaction and the propagation of localised effects to citywide or regional effects through global pipelines and local buzz (Bathelt et al., 2004; Zhou et al., 2011) or scaling-up processes (Wei et al., 2007), thus increasing the spatial range for people to choose their places of employment.

Lastly, HCS crystallises unevenness or inequality in space; therefore, special attention should be given to inclusive growth in a human-centred era. With the increasingly significant role of HC, there might be rising spatial inequality. Alongside the fast growth and emergence of a knowledge economy, wage inequalities in Chinese cities may increase despite government expenditure on social welfare and public employment (Liu et al., 2020). In Los Angeles, inequalities embedded in socio-spatial relations are entrenched in urban schooling (Lois, 2007). In Milan, although there are general criteria for universal access and equality, socioeconomic inequalities around schooling are still implied (Cordini et al., 2019). The mechanism countering the inequalities associated with HC must be considered given its compounding effect and intergenerational feedback on people–spatial development.

Together with social, economic, and spatial externalities, HC increasingly and endogenously affects spatial development. This has been illustrated in knowledge economies, a wide spectrum of industries and region–industry effects (Liu, 2014; Morris et al., 2020), regional knowledge capabilities based on institutions, and firms open to innovations in the new era of globalisation (Cooke, 2005). Tandon et al. (2016) show that it is inappropriate to view HC as only relevant to high-technology industries and information and communication technology companies. Knowledgeable and experienced employees support learning and empower firms to acquire, develop, transfer, and manage knowledge-related assets, thereby elevating the knowledge management process (Seleim et al., 2007; Cohen and Olsen, 2015), which is essential for resource-based integration (Marrocu and Paci, 2012). Such an advantage can be translated into regional development. Extending the concept of HC to facilitate a spatial transition to a knowledge-based economy, characterised by the creation, dissemination, and use of knowledge to enhance its growth and development, becomes meaningful. However, presently, there is fragmented knowledge about HC as an endogenous factor of spatial development.

HCS provides some clues, from a geographical perspective, to understanding the interplay and reciprocity of people and space. Knowledge-based creative cities and shrinking cities face very different situations, which may compound the results of the organic interplay of various types of HCSs. Endeavours of knowledge-based creative cities are generally described in terms such as ‘smarter’, ‘creative class’, ‘attractiveness’, and ‘the quality of place’ (Lee, 2014; Escalona-Orcao et al., 2018; Basle, 2021), all of which shape HCES and require HCRS. However, with dramatic demographic changes, interests have expanded from addressing shortages in skills and expertise to shrinking populations. Ageing and population contraction are increasingly reported in Europe, the United States, the United Kingdom, Japan, and China (Oswalt and Rieniets, 2006; Martinez-Fernandez et al., 2012; Rhodes and Russo, 2013; Haase et al., 2016). It is perhaps a long-term outcome of uneven spatial development and ongoing and future demographic changes such as lower fertility (Nash, 1994), which places a cloud over urban development with insufficient labour supplies, decreasing consumption power, abandoned land, and social instability (Yang and Dunford, 2018). It has become imperative for many cities and regions to think about ways to attract and retain people or, in other words, gain HC. Although cities may benefit from the labour attracted by local universities, there are some exceptions. For instance, approximately 90% of college students leave Wuhan in China, as they do not want to work in the city because of the lower salaries and higher living costs compared to other cities. Moreover, living and working conditions fall short of expectations (according to the authors’ investigation/interview with the Wuhan local government in 2004). Other cases of a mismatch between HCES and HCCS include Pittsburgh and Cleveland; educated youth are moving out of these cities (Hansen et al., 2003; Gottlieb, 2011). This shows that cities do not necessarily harvest the profits of HC cultivation. In Sweden, migration rates are the highest among young adults, especially students, and their location choices affect the regional distribution of HC, growth, and local public sector budgets (Berck et al., 2016).

The interplay of various types of HCSs drives the internal dynamics of cities and regions. The pursuit of income, education, and natural and social environs motivates people to move to places with higher HC, thus resulting in a displacement of intellectual assets in the modern economy (Tandon et al., 2016). As Rutten (2017: p. 159) points out, ‘knowledge creation is recognised as interaction between individuals in a social context, but geography-of-knowledge-creation research inadequately connects social context to physical place’. HCS provides an approach to enable conversations pertaining to economic and human development in particular social contexts by considering the reciprocity of PERs and the organic linkages between investment and rewards in the accumulation of HC; this helps develop typologies connected to physical places for these conversations.

HCES is important for a place to carry out technological innovation (Romer, 1990; Danquah and Amankwah-Amoah, 2017) and to facilitate technology catch-up in the 21st century (Lee, 2013). Its potential may lie not only in enhancing productivity but also in improving the quality of industries by increasing firms’ ability to develop business ideas and innovate (Nieves and Quintana, 2016), fostering start-ups and stimulating the growth of small and medium-sized enterprises (Jansen et al., 2013), and enabling governments to initiate and implement policies more effectively (Danquah and Amankwah-Amoah, 2017). Recent studies show that the geography of urban high-tech industries is primarily based on the scale of existing high-tech activity and the size and extent of metro areas (Adler and Florida, 2021)—in other words, the level of HCES and citywide or regional multiplayers.

Unlike economics or business management studies that measure HC in business operations, HCS is devoted to measuring HC and its supportive settings at the spatial scale to identify spaces to cater to people’s needs and enhance their skills, commitment, and productivity. HCS constructs a premise to develop certain types of industries. China’s and India’s remarkable growth should be attributed to their growing pool of well-educated and skilled people (Florida et al., 2012; Fu and Gabriel, 2012; Singh and Nayak, 2016) and the continued growth of HC that relies on gradually developed HCSs, thus allowing them to enter the global economy and reap the benefits.

## Human capital in space: selection, sorting, and matching

In space, it is always fascinating to ask why HC in some places is higher than that in others and why places differ in terms of their loss and gain of HC. As HC is largely intangible and embodied by people, these questions can be investigated using a selection-sorting mechanism between people and space.

Selection indicates that particular residences and workplaces contain people with a certain type and level of HC. In fact, society develops at different paces and people choose favourable places to cater to their needs. Mobility is a prominent feature of talented and skilled people (OECD, 2008), typified by an agglomeration process of HC in spaces. Just as agglomeration economies are often proximally located by a group of firms sharing similarities and various types of relations, certain types of HC are centred in particular places, manifested as a group of people engaged in similar cultivation, employment, or refreshing activities. The agglomeration of HC emphasises its spatial structure. It denotes a space that enjoys a higher HC level. Similarly, there are diffusion and spatial spillovers of HC as people explore new places, which are in turn constructed for people.

Beyond spatial attributes, the factors influencing people’s selection include individual characteristics and the extent of people’s knowledge about the place, which is dependent on HC. Thus, the spatial selection of HC is endogenous. People with a higher level of HC enjoy a wider spatial reach. Exogenous factors may include certain institutions and regulations for school catchments. Notably, not all selections are successful or static; people may leave the place they choose for many reasons. If they are ill-informed or misguided, the space will not meet their expectations. Normally, big cities are more attractive than small ones, and young graduates are more likely to flow into big cities. However, they may move to small cities if they cannot afford the high living costs or adapt themselves to competitive environs with high-level HC under, perhaps, the so-called survival-of-the-fittest mechanism. This situation somewhat explains the high inflow and outflow of people as well as job changes in high-level HCES, such as big cities and high-tech industry parks. After achieving their goal, people may stop their selection. For instance, people may relocate after their children have entered or graduated from school, which explains the high turnover of houses in school catchments. These attributes highlight and consolidate the self-selection of HC in space. Baldwin and Okubo (2006) note that the skill premium is an increasing function of the number of high-skilled workers in a region.

HC is sorted in space, with places occupied by people who have the desired HC for the location. Selection refers to people selecting places, whereas sorting occurs after people’s selection. People may want to choose a certain place but be required to leave due to various reasons such as high living costs and poor employment opportunities. Brakman et al. (2021) identified three types of skills—education, sector, and occupation—to examine their role in sorting people in China’s large cities. Interestingly, high- and low-skilled workers can be attracted to large cities (Eeckhout et al., 2014), where high skills are the main attraction of the place, while low-skilled workers play a supportive role. Space sorting stems from the heterogeneity of space and HC. In the interplay, the highest level of HC moves to the spatial core, while the lowest moves to the periphery.

The sorting process for HC can be collective action. As people use different spaces at different times, different types of HCSs work together in HC sorting. Big cities have more allure because, in addition to high wages, people can enjoy a higher quality of life and their children can receive a better education. In the US, students often move to a new area to attend college and then stay there, inducing an accumulation of an educated population in that area (Winters, 2011). In Colombia, talented individuals move to big cities to attend college and remain there for work; individuals who move to smaller cities have lesser abilities than those in college cities (Bacolod et al., 2021). In cities, skill-based sorting acts as a driver of urban stratification, given the interplay of parental cognitive skills and metropolitan opportunity structures, with race, income, education, housing market conditions, and spatial proximity all having an influence (Clark and Maas, 2012; Schachner and Sampson, 2020). In China, the urban environment sorts residential choices (Zhu et al., 2022).

Underlying the collective sorting are the progressive and interactive processes of human movement in socioeconomic and physical spaces. Clearly, HC exists in tandem with the wealth creation process because individuals, families, cities, and regions must invest in HC. In return, this can be converted into wealth for the city. As Mincer (1989: p. 3) argues, ‘A more rapid pace of technological progress should induce increased inputs of HC, formed at school and on the job, by making their acquisition more profitable. Both utilisation and wage effects ought to be observable’. HC not only complements the other forms of capital to facilitate production but also links income, a city’s level of wealth, and regional development. Thus, HC improvements, regional wealth creation, and income increases all occur with investment in education, healthcare, and recreation. Many studies have demonstrated that the lack of high-skilled jobs contributes to the out-migration of educated youth from rural areas (Huang et al., 2002) because the economic return on education is higher in big cities—about 5.4% compared with less than 1% in rural or smaller cities in China (Wang et al., 2020). Declining populations and dwindling tax bases make it increasingly difficult for rural communities to deliver public services efficiently (Artz and Yu, 2011), which is a virtuous cycle for spatial investment and HC accumulation.

The sorting of high-skilled workers is often advanced as a source of spatial disparity in economic outcomes (Ahlin et al., 2018). During 1986–2008, 45% of the increase in wage disparities in Sweden was due to the sorting of workers by cognitive and non-cognitive skills (Hakanson et al., 2021). Through migration and mobility, the sorting process contributes to widening health inequalities in the population (Darlington-Pollock and Norman, 2020) and spatial segregation in neighbourhoods (Cordini et al., 2019). Schachner and Sampson (2020) argue that urban studies should examine skill-based sorting as a driver of stratification, as they show that upper- and upper-middle-class parents predict sorting in average public school test scores rather than in neighbourhood socioeconomic status. More critically, negative sorting may occur in an area represented by a large pool of low‐skilled labour—poor infrastructure and less‐advanced technology—because the returns on skills are lower in industries with strong production linkages due to the substantial deterioration in quality when the size of the production linkages increases, as found in India (Asuyama, 2019).

Spatial matching stems from the interplay between spatial selection and sorting, highlighting that HCS and HC can support each other. Scholars measure efficiency or optimal matching between industries and cities from a utility perspective (Helsley and Strange, 2014). Accordingly, larger and thicker labour markets can improve the quality of the match between firms and skill attributes, which increases competition in the matching process (Venables, 2011). Spatial matching can increase cooperation opportunities between firms and researchers located nearby, although success is not guaranteed (Calcagnini et al., 2016). Notably, matching opportunities are significantly determined by knowledge exchange (Berliant et al., 2006). In a larger and denser labour market, such as in the science and technology, engineering, and mathematics industries in the US, the probabilities of matching can rise (Wright et al., 2017).

## Dynamic capabilities and investment

The previous sections discussed the internal rewards of HC for people and space. The role of HC in fostering spatial development is not static and acts as an endogenous power to attract and leverage external resources, which can be called dynamic capabilities. Through various selection-sorting processes and intergenerational transmissions, HCS provides an approach to investigate dynamic capacity spatially. People with higher levels of knowledge, skills, and experiences are capable of identifying potential opportunities and threats (McKelvie et al., 2009), adapting to new circumstances, and integrating, reconfiguring, and reallocating resources and capabilities (Teece, 2009). In terms of place, dynamic capacity refers to resource integration and reconfiguration ability of a region to respond rapidly to fluctuating conditions (Teece, 2012; Singh and Rao, 2016) to create, extend, or modify its resource base, seize opportunities, and achieve new resource configuration. With increasing competition during the ongoing financial crisis, dynamic capabilities have become an indispensable element in the success of regions and one of the strategic driving forces for elevating performance and sustaining competitiveness.

Specifically, integration capability refers to the capacity of a place to determine the value of its existing resources and integrate them to develop a new resource base and capabilities. In contrast to production dominating the regime of research in a city and regional growth, HCS distinctively integrates the lifespan of individuals and households with space, which brings opportunities and potential for variety in the interactions among different types of HCSs. Reconfiguration capability refers to the recombination and transformation of existing resources and assets to empower a region to acclimatise to fluctuating market conditions. HC matters in both types of capabilities, and it is difficult to distinguish between them because they are interrelated and interchangeable during both HC and regional development. This is because HC provides additional inputs to create, apply, and transfer newly acquired knowledge (Argote et al., 2003) and supports the renewal of the resource base that has a bearing on the dynamic capabilities for development. In China, for instance, HC enables regions to transform from being recipients of external resources to active contributors in the global market (Yang et al., 2021).

Owing to the decreasing return of HC and competition among spaces, there is a need to invest in and capitalise HCS. Intergenerational financing and on-the-job training have been recommended to avoid such decreasing effects (Schultz, 1961). However, the spatial differences between individuals and public inputs to HC formation are unclear. Investment in most HCSs is perhaps ‘patient capital’, especially in the case of education and healthcare. As observed, industrial investment, physical infrastructure, and digitalisation have already contributed to uneven global development over previous decades (Aryee et al., 2013); HC investment is an important vector with even more far-reaching influences. During the COVID-19 outbreak, cities and regions with insufficient investment in public health services faced enormous challenges that may have exacerbated the pressure on HC accumulation and uneven spatial distribution. Demand for high-quality schools reflects the scarcity of educational resources, which results in social inequality and spatial segregation.

Demographists have proposed intergenerational financing models to justify monetary inputs as a medium for generations to continuously improve education or HC (Michel and Vidal, 2000). As such, spatial investment is a conductor that actively responds to parental intergenerational financing to more efficiently accumulate HC and foster positive feedback for PERs spatially, rather than focusing only on attracting talent. Meanwhile, spatial justice is imperative for considering not only talent but all people. Additionally, studies have investigated whether universities can directly support industrial innovation (Fallah et al., 2014) even if they cannot finance their research budget through licensing alone (Miyata and Shavinina, 2003). Therefore, public funds are required to maintain high-quality research and HC outputs.

## Conclusions

People, as active actors, are crucial to the creation and transfer of wealth, assets, and knowledge from one generation to another and from one place to another. Therefore, it is important to focus on people to more sufficiently appreciate current and future trends related to human development. This article focuses on the linkage between people and spatial development by introducing and promoting the idea of HCS. It has some contributions and implications.

First, by proposing HCS, this article establishes a bridge to promote dialogues, especially regarding the interactions among population, labour, economic geographies, and other social issues. It sets up an arena to further theorise newly proposed ideas—for instance, exploring the human and spatial implications of health-selective migration in new paradigm mobilities (Darlington-Pollock and Peters, 2021); the synergy between knowledge and creative economies, talent, and amenities; and the geographies of contemporary educational restructuring (Thiem, 2009), especially through the processes of *jiaoyufication* (Wu et al., 2018) and studentification (He, 2015). It also encourages better engagement of geographers in popular debates to include demographic trends in development and the related issue of shrinking cities and regions. Rutten (2017) proposed ‘conversations’ between social spaces of knowledge creation and the physical space of attractiveness of places. HCS may extend from this to consider a lifespan of human needs to construct an endogenous power of long-term human–spatial development. A closer examination of the subject using HCS may provide a new outlook for investigating interactions between people and the economy in geographical spaces where there are differences in intergenerational financing and territorial wealth enhancement. However, HCS is much more contextualised. The relative importance of the three types of HCSs may differ per the economic stages of cities and regions. It is also affected by the social environment; for instance, ethnic issues may influence HCS more in the US than in China. Moreover, some types of HCS may not exist in some cities (e.g., university-based HCCS). Thus, we must contextualise HCS by linking it with the economic functions of spaces and their social and cultural backgrounds. Further elaborations on the HCS concept could be an interesting future research direction.

Second, the article provides clues and insights for understanding how space is structured by human development. While Crevoisier and Rime (2021) establish an insightful typology of urban income flows and activities to shape urban competitiveness and attractiveness for both local and external consumers, the work fails to capture the drivers and impact of human and spatial development. HCS thus provides a new approach for examining why cities and regions should be structured by and for humans; in particular, future research can contribute further insights into the spatial selection and sorting of HC. However, HCS investment is expensive, and spatial matching between people and space is not always perfect. Moreover, an in-depth understanding of leveraging the matching mechanism is necessary.

Finally, this article implies that HCS is a crucial issue that requires careful and systematic policy design and capital investment to bolster long-term energy (mainly in cities and regions), particularly creating endogenous power for human and spatial development. Compared to traditional accounting for physical capital, firms, and associated production processes, this article encourages a people-oriented shift, not only in terms of its emphasis on endogenous economic growth but also by paying more attention to the quality of places in terms of amenities and creating synergies between people, places, and economies. Although the economic rewards are not instant, HCS can dramatically affect the dynamic capacity of places.

Admittedly, there are limitations and many challenges to incorporating HCS into human geography and exploring PERs. First, there is lack of data on the systematic documentation of the various statuses and levels of HC at different spatial scales, especially for cities and regions. Different types of HC may imply differences in knowledge, skills, and development potentials. In many cases, empirical studies use education data owing mainly to their availability, while some important features, such as health status, are less frequently investigated. The Jorgenson–Fraumeni (1992) approach considers education and income together but requires micro- and macro-data. Considering the availability of data, it is challenging to widely adopt this approach, especially for systematic research on a large set of cities and regions. Lutz et al. (2021) estimated skill-adjusted HC worldwide and noted a bias for many countries if it is measured by considering education alone. Consequently, research results are inconsistent and difficult to compare. There is thus a need to design a methodology for measuring HC and HCS in geographical studies. Recent night light index-based applications illustrate new area-based estimations (Yang and Pan, 2020a), which can be referenced to facilitate the methodological dialogue between sociological and geographical studies. Second, humans develop with various needs, and, accordingly, HCS is not exclusive, as it changes and upgrades with social, economic, and spatial development. There is a long way to go to make HCS more detailed. Third, this article does not deny the importance of other forms of capital (Bourdieu, 1986) but attempts to encourage greater consideration of HC, as it builds a platform for conversations between social and physical spaces. The physical and social interplay between HC and other forms of capital could be a future research topic. Analyses of HCS need further elaboration in more physical and wider spaces, such as employment areas, with a network and accessibility analysis (Giuliano et al., 2012). Finally, as spatial inequality and uneven development may be further stimulated by HC accumulation and agglomeration, ‘patient’ investment, waiting for both economic and non-economic rewards that may not be instantly realised, is strongly encouraged.

## Data Availability

Data sharing is not applicable to this article as no datasets were generated or analysed during the current study.
